# Membrane protein structure and dynamics probed by MicroED

**DOI:** 10.1042/BST20250098

**Published:** 2026-05-08

**Authors:** Orel Paz, Tamir Gonen

**Affiliations:** 1 Department of Biological Chemistry, University of California Los Angeles, Los Angeles CA 90095, U.S.A.; 2 Molecular Biology Institute, University of California Los Angeles, Los Angeles, CA 90095, U.S.A.; 3 Department of Physiology, University of California Los Angeles, Los Angeles, CA 90095, U.S.A.; 4 Howard Hughes Medical Institute, University of California Los Angeles, Los Angeles CA 90095, U.S.A.

**Keywords:** cryoEM, crystal, membrane proteins, microcrystal electron diffraction, MicroED

## Abstract

Membrane proteins are essential to cellular signaling, transport, and homeostasis, yet their amphipathic nature, dependence on lipids or detergents, and typically difficult expression and purification make them difficult targets for structural methods such as X-ray crystallography. Moreover, most membrane proteins in the human proteome are too small for investigation by single-particle electron cryomicroscopy methods. Microcrystal Electron Diffraction (MicroED) has emerged as a powerful method for overcoming these barriers, allowing structure determination from nanocrystals of membrane proteins embedded in the near-native environment of the lipid membrane. In the present review, we discuss how recent improvements in MicroED, such as focused ion-beam milling and high-throughput data collection approaches, facilitate structure determination and investigation of protein dynamics. We focus on applications involving junction-forming proteins, G protein-coupled receptors, and ion channels, where MicroED has revealed physiologically relevant assemblies, lipid interactions, and transient functional states that were not attainable by other structural biology methods.

## Introduction: membrane proteins pose unique challenges

Membrane proteins are central to many physiological processes, including signaling and cellular homeostasis. Within the lipid bilayer, they function as receptors, channels, transporters, and enzymes. While only ∼30% of the human genome encodes membrane proteins, they are the largest target of all pharmaceuticals [1]. More than one-third of all pharmaceuticals on the market target one class of membrane proteins, the G protein-coupled receptors (GPCRs) alone [2]. Despite their importance, experimental structure determination of membrane proteins remains scarce, with far fewer structures reported than for soluble proteins [3].

Membrane proteins pose particular challenges for experimental structure determination. Despite remarkable advances in structure prediction such as AlphaFold [4], membrane proteins remain a particular challenge, mainly because their folds depend on lipid environment and conformational states not directly modeled by current algorithms [5]. Membrane proteins are amphipathic, possessing hydrophobic transmembrane regions and hydrophilic domains. These biochemical properties make expression, purification, and crystallization challenging at every stage. Moreover, membrane proteins typically have extreme isoelectric points, either very low or very high, which further complicates their solubility in common buffers. Finally, many membrane proteins require native lipids for their function, and those are frequently lost during purification, often leading to assemblies that may not represent the native state of the protein [6]. The amount of attainable protein is often low when compared with soluble proteins. Given these challenges, studies using single-crystal X-ray diffraction (SCXRD) often fail, as large crystals and large amounts of stable proteins are needed for these experiments.

In an effort to accelerate membrane protein structure determination, the electron cryomicroscopy (cryoEM) method, single-particle analysis (SPA), has been widely employed [7–9]. Here, crystals are not necessary, but rather individual particles are imaged directly under cryogenic conditions. A stable homogeneous preparation is still required for high resolution, and grid preparation requires optimization, but at least the crystallization step is bypassed [7,8]. SPA has also been used to investigate conformational heterogeneity and protein dynamics through classification of large particle datasets and reconstruction of multiple structural states. However, this approach is only viable for a limited portion of the membrane proteome, as the vast majority of membrane proteins are far below the size limitation afforded by SPA. In fact, the average size of a membrane protein in the human proteome is merely ∼35 kDa [10,11], which is not attainable by SPA [12,13].

**Figure 1:**
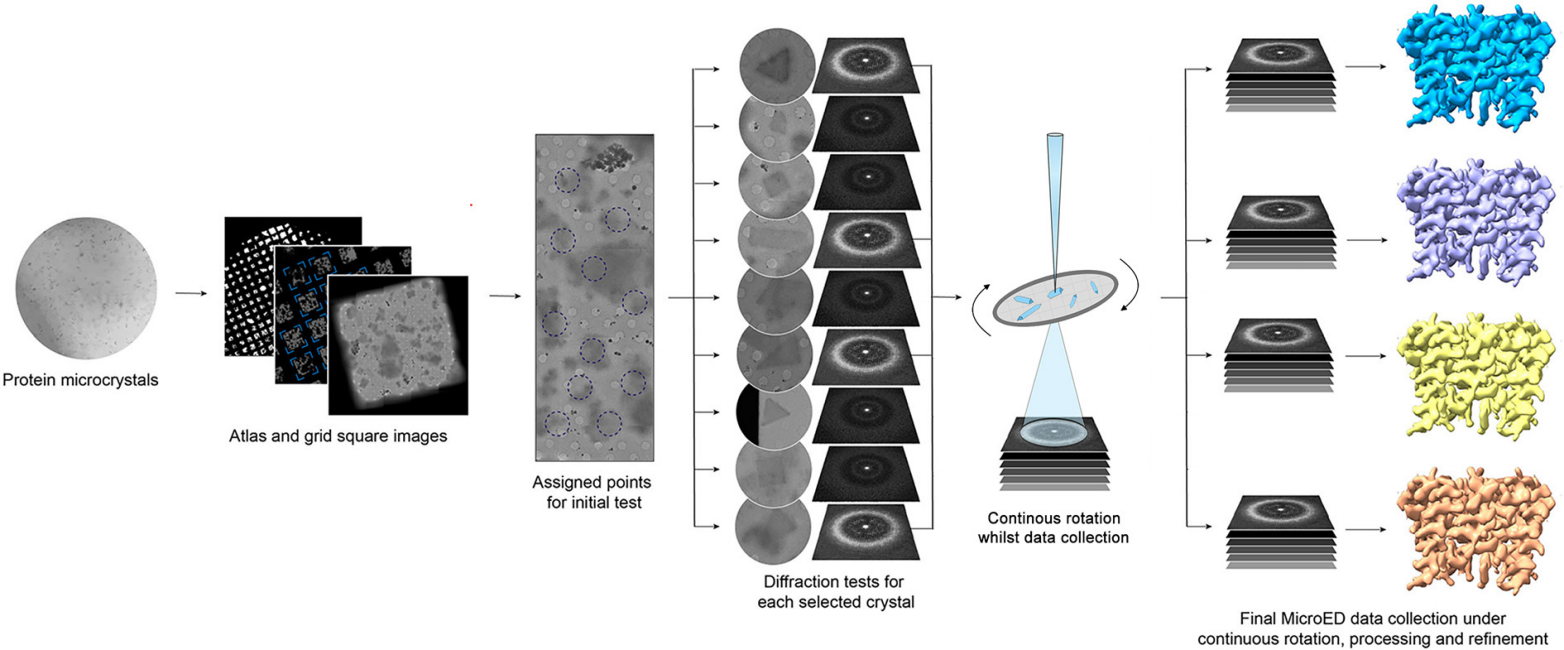
Workflow of high-throughput MicroED data collection Microcrystals are applied and vitrified on cryo-EM grids. Grids are viewed in low magnification to identify nanocrystals. Candidates are screened by a test diffraction, and those that show high-quality diffraction are then subjected to data collection by continuous rotation. Then datasets are processed and further refined to obtain final structures.

Microcrystal Electron Diffraction (MicroED) is a different cryoEM method in which electron diffraction is collected from crystals a billionth the size that is required for SCXRD (Figure 1) [14,15]. Membrane protein crystals are prepared either after reconstitution into lipid membranes (vesicles, sheets, and lipidic cubic phases (LCPs)) or in detergent [6,16]. Because the crystals that are used for MicroED are so small, a vanishingly small amount of material is also needed for crystal growth. Moreover, MicroED does not have a size limitation as SPA does: in fact, there is no upper or lower limit that has been encountered thus far in MicroED experiments, and even single amino acids have been studied successfully at atomic resolution [17,18]. In the present review, we highlight several examples of membrane protein structures that were not attainable by SCXRD or SPA, but MicroED delivered experimental structures. We also discuss cases where high-throughput methods in MicroED led to visualization of protein dynamics using experimental maps. Finally, we provide an outlook for the future of MicroED in studying challenging membrane protein targets for pharmacological development.

## MicroED: basics and methodology

MicroED is a cryo-EM method in which electron diffraction data are collected from crystals that are micron or submicron in size using a typical transmission electron microscope (TEM) [14] (Figure 1). The method might be thought of as a cross between electron microscopy and X-ray crystallography: it uses electrons rather than X-rays, but both rely on diffraction data from crystalline samples. Electrons interact with matter much more strongly than X-rays, allowing even tiny crystals to produce measurable diffraction [19]. Furthermore, electrons are sensitive to the Coulomb potential (electric charge distribution) in the crystal rather than the electron density [20]. This means that while SCXRD delivers an electron density map, MicroED delivers a charge density map in which charges, ions, and protons are easily discernible [20]. For instance, early MicroED research found that Coulomb potential maps at ∼3 Å resolution could already reveal the positions of hydrogens [18,21] and the charged states of metal ions in proteins and amino acid side chains [20], such as the divalent state of Ca^2+^ bound in a Ca-ATPase pump [20] and the protonation of acidic residues.

MicroED data collection involves cryo-cooling the microcrystals and exposing them to a low-dose electron beam while continuously rotating the crystal during the exposure (Figure 1) [15,22]. Similar to the rotation method in SCXRD [23], diffraction data are captured as a continuous rotation movie [24]. There is an important interplay between signal and radiation damage: since electrons cause damage quickly, relatively low electron doses are utilized for MicroED, which in turn leads to weak signals [15,25]. Each crystal is usually rotated by at least 20 degrees to allow indexing without prior knowledge of unit cell dimensions or symmetry [22,26], and usually data from multiple crystals (or multiple regions of one crystal) are merged to achieve a complete dataset [27]. The resulting diffraction intensities are processed with standard crystallographic software to index reflections and determine structure factor amplitudes. MicroED is unique among electron diffraction approaches in that its data can be processed using standard crystallographic software. This is made possible by continuous-rotation data collection [26]. Phasing the MicroED data can then be accomplished by molecular replacement (often with homologous structures) or, in the case of high-resolution data, by direct methods or *ab initio* approaches [28]. Notably, molecular replacement was initially established in electron diffraction using 2D crystals of aquaporin-0 (AQP0), where phases from a homolog (AQP1) were joined with electron diffraction amplitudes to solve the novel AQP0 structure at atomic resolution [29].

Preparing samples for MicroED is a crucial step, especially for membrane proteins. Typically, specialized crystallization methods such as LCP [30] or bicelles [31] are used to provide a native-like lipid environment for membrane proteins [32]. Alternatively, membrane proteins can be incorporated into membrane vesicles to form crystalline sheets suitable for electron diffraction [16]. The microcrystal solution is dispensed onto a TEM grid, which is typically a carbon grid with holes that has been glow-discharged to make it clean and slightly hydrophilic [24]. Excess liquid is blotted away (often from the reverse side of the grid) to leave crystals adherent in a thin film. To vitrify the sample and maintain the crystals at cryogenic temperatures, the grid is next plunge-frozen in liquid ethane (∼90 K). If crystals are thicker than ∼0.5 μm or are embedded in viscous media, cryo-focused ion beam milling (cryo-FIB) can be used to thin them to the appropriate thickness [32,33]. Using a focused ion beam, one can shave a frozen crystal down to a thin lamella of ideally 150–300 nm thickness [32,33]. For instance, scanning electron microscopy can be used to identify big plate-like crystals developed in LCP [30,34] or bicelles in the frozen sample; the FIB then carves out a window around the crystal and gradually grinds it to the appropriate thickness [33]. This procedure has been improved by the development of plasma FIB, which uses inert gas ions like xenon or argon to mill biological material more evenly and with less damage than conventional gallium ions. Importantly, it was reported that xenon and argon can mill through the viscous lipid membrane in LCP, while gallium only does so after depositing high energy, which destroys the crystallinity of the sample [30]. An integrated fluorescence light microscope can be used to locate protein crystals grown in LCP and correlate light-EM for milling. This was demonstrated for several recent examples [27, 30, 33, 34], with a detailed protocol published [33]. After FIB milling, the thin crystalline lamellae are transferred into the TEM for data collection.

MicroED data acquisition involves slowly rotating the stage containing the crystal while recording diffraction images with a fast camera [26]. Because the stage rotates during the exposure, each frame in the movie contains a wedge in reciprocal space, which in turn allows for indexing without prior knowledge of unit cell dimensions or symmetry [24], as those can be determined experimentally if at least 20 degrees are collected per crystal [22,26,35]. High-resolution reflections can be obtained from incredibly small crystalline volumes due to the strong interactions of electrons with atoms; for example, a GPCR structure was solved by MicroED using a total crystal volume of less than 1 μm^3^ [33]. Similar studies for the same sample using SCXRD or serial femtosecond crystallography (SFX) used 30 and 10 µm^3^ of crystalline material to reach the same resolution as MicroED [33]. Importantly, while SCXRD and SFX used a lot of samples, even hundreds of milliliters full of large crystals, MicroED only required a single crystal to deliver the same resolution structure.

Samples in MicroED are kept in a frozen hydrated state under cryogenic conditions to deliver more accurate reflections and help mitigate the effects of radiation damage [36,37]. However, radiation damage accumulates quickly; typically, a total exposure on the order of only ∼1 e⁻/Å^2^ is sufficient to damage disulfide bridges, deteriorate protein features, and limit the attainable resolution [36]. For these reasons, fast and sensitive cameras are typically employed, enabling complete data collection at total exposures of less than 1 e⁻/Å^2^ and allowing structures to be determined at atomic resolution [17,37,38]. Even so, depending on the dosage of collection, diffraction intensity could fade as the crystal is rotated, and merging data from multiple crystals is a common way to solve the structure, leading to high completeness and quality [25,36]. A recent report details the gold standards for a MicroED experiment [39].

**Figure 2:**
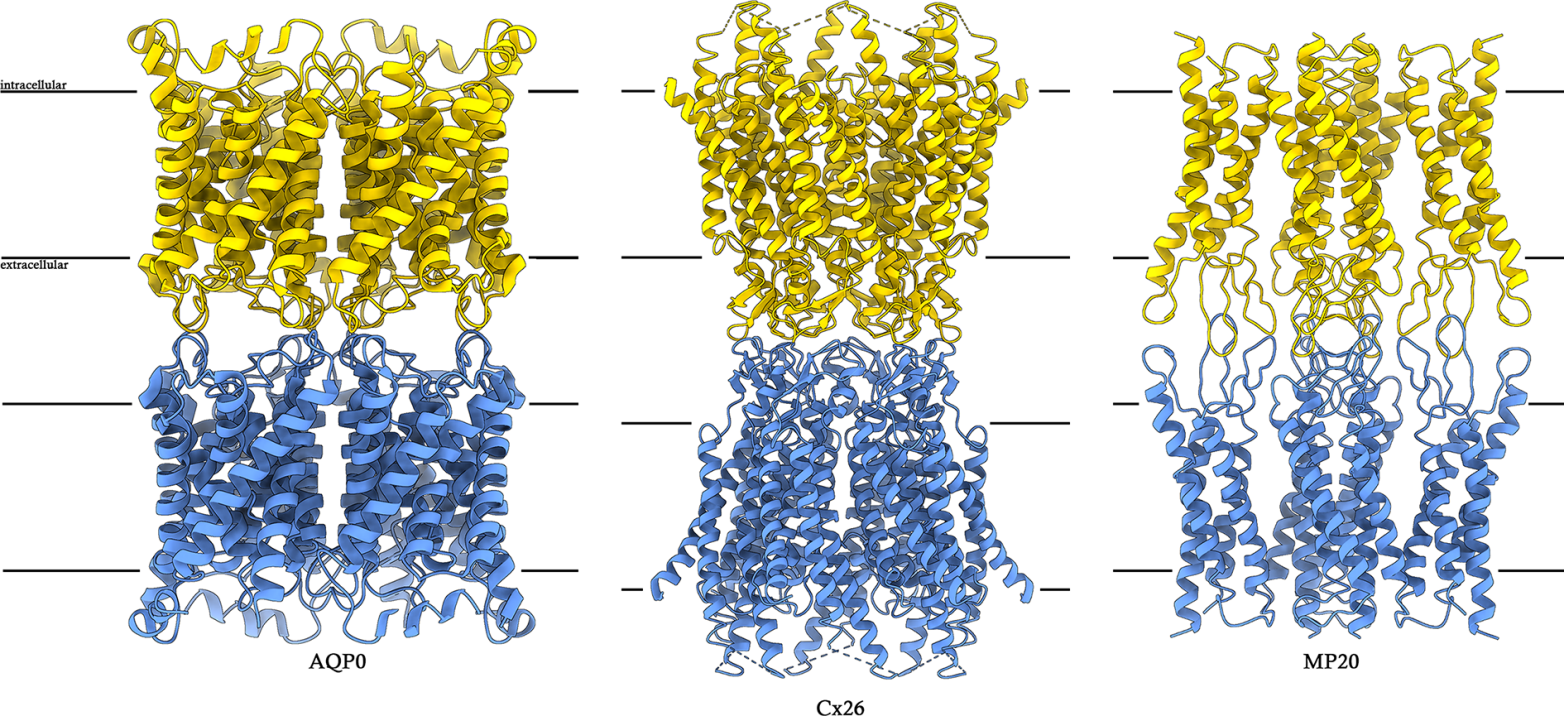
Junction-forming membrane proteins Representative examples of junction-forming membrane proteins that were solved by electron diffraction. Adhesive cell-to-cell junction of the water channel aquaporin-0 (left). Communicating cell-to-cell junction of connexin 26 (middle). Adhesive cell-to-cell tight junctions of MP20, a distant member of the claudin family, determined by MicroED. In all cases the presence of a lipid membrane during crystallization was critical to obtaining the physiologically relevant structure of the junction.

**Figure 3:**
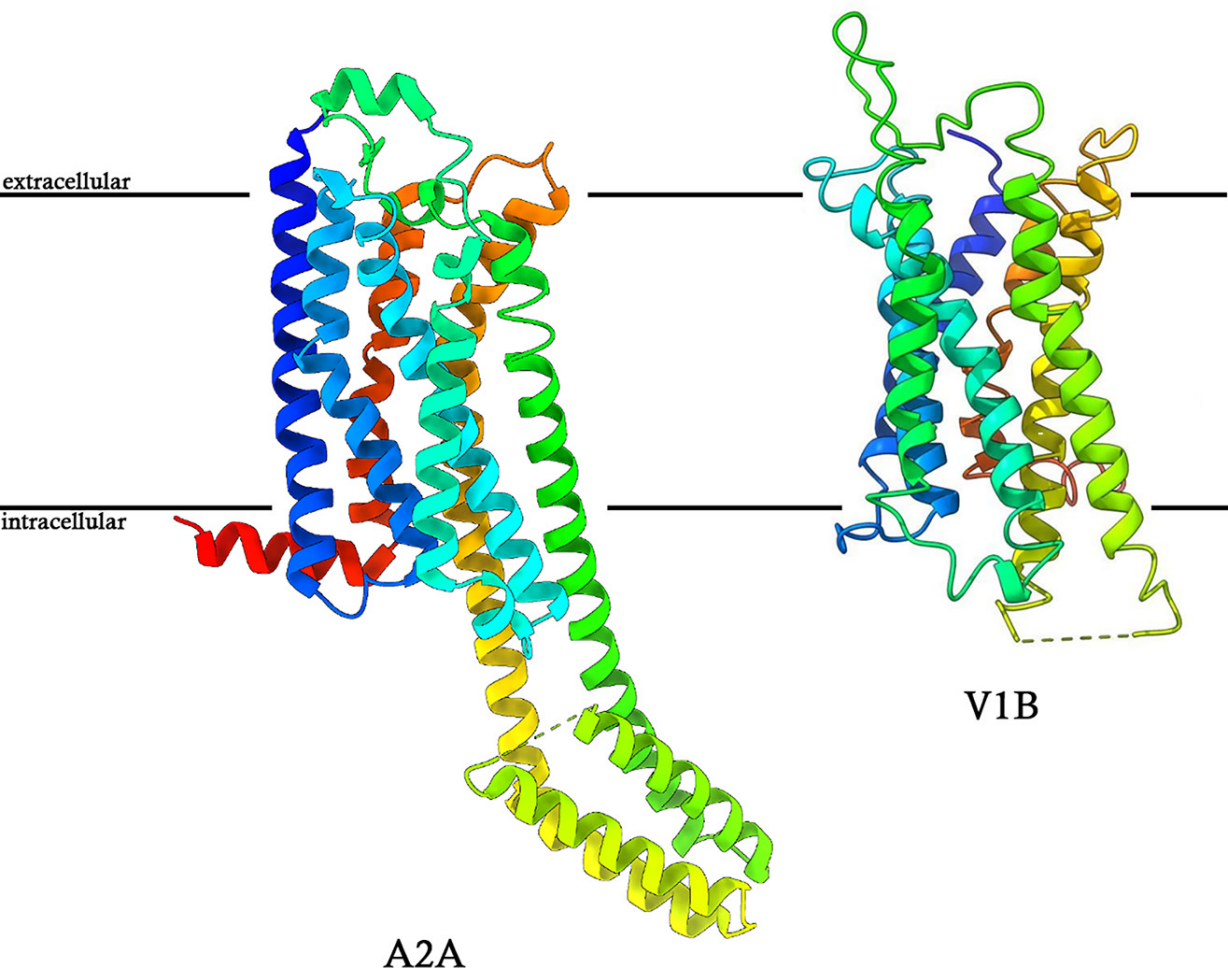
MicroED structures of human GPCRs Examples of GPCR structures determined by MicroED. Left, the human adenosine A2a receptor was determined from a single nanocrystal only 200 nm in thickness to 2.0 Å resolution [30]. Right, the novel structure of the human vasopressin 1B receptor [34]. Structures are colored in rainbow with the N-terminus in blue and C-terminus in red.

**Figure 4:**
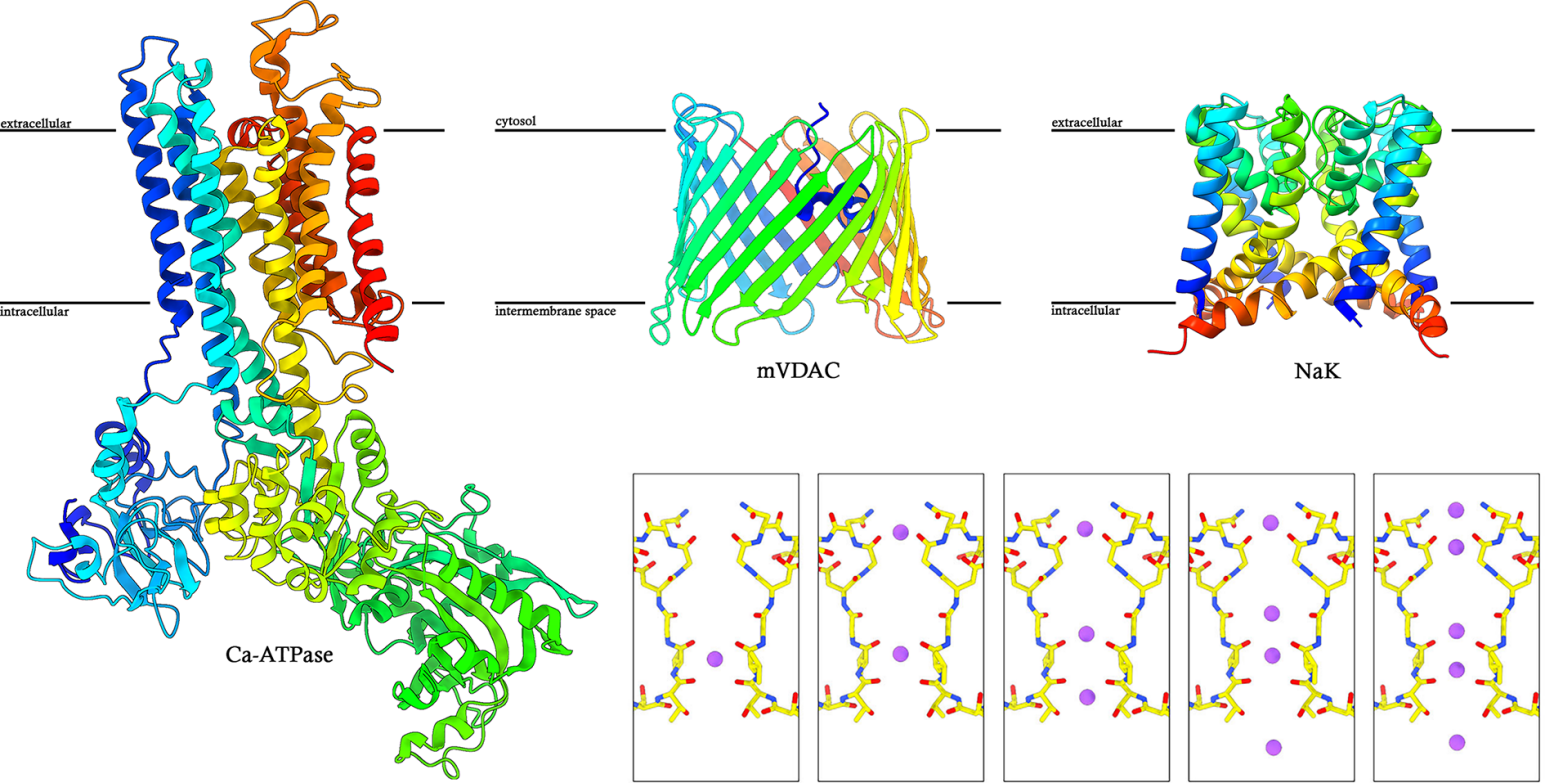
MicroED structures of ion channels and channel dynamics Examples of ion channels solved by MicroED. Left, Ca-ATPase [20]; middle, mVDAC—a functional mutant that resisted structure determination by other methods [41]; and right, the nonselective sodium channel NaK [46]. Inset, high-throughput MicroED pipelines were used to image and capture distinct functional states in NaK, showcasing the use of MicroED in determining channel dynamics [42].

Despite these challenges, MicroED has emerged as a viable method for determining the structures of membrane proteins that were not attainable by any other structural biology method [34, 40–42] and achieved impressive resolutions in several studies where ions, ligands, activators, inhibitors, protonation state, and even hydrogen-bond networks in some cases were visualized experimentally [17, 18, 42–45]. Below we outline several of these studies focusing on junction-forming membrane proteins (Figure 2), receptors (Figure 3), and ion channels as examples (Figure 4).

## Junction-forming membrane proteins

Some membrane proteins specialize in the formation of cell–cell adhesion. These proteins hold two cells in close apposition, sometimes creating a tight seal that is impermeable even to simple diffusion of solutes (such as tight junctions [47,48]); some form cell-to-cell adhesive junctions that still allow diffusion through the extracellular space [49], while others form cell–cell communicating junctions, creating a continuous soluble path between apposing cells for solute exchange [50–52]. Representative members of these classes of adhesion proteins were determined experimentally by electron diffraction, and for some, only electron diffraction delivered the physiologically relevant assembly [27,29].

AQP0, also known as the major intrinsic polypeptide or MIP, is a water channel in the fiber cell membranes of the eye lens, where it is known to form adhesive 11 nm thin cell–cell junctions [53,54]. Aquaporins typically form a highly efficient path for water permeation through biological membranes [54]. The first structure of an aquaporin, that of aquaporin-1, was determined by electron crystallography of 2D crystals [55,56]. Nearly a decade later, the structure of AQP0 was determined by electron diffraction using double-layered 2D crystals [29]. In both cases, the protein formed a tetramer, which was crystallized within the native environment of a lipid membrane and adopted the canonical structure of an aquaporin, namely, six TM helices with two half TM helices containing the NPA motif, which is important for proton exclusion [29, 53–56]. However, in the case of AQP0, the protein also adopted an unexpected structure when it was crystallized in the lipid bilayer: it was found to mediate adhesive junctions between two membranes with one AQP0 tetramer engaging an AQP0 tetramer from the apposing bilayer (Figure 2).

Since AQP0 was crystallized in double-layer membranes, it represents the first example of a membrane protein determined by electron diffraction from very thin 3D crystals; crystals only two membrane layers in thickness [29]. The crystals, which were several micrometers in length and width, were only 160 Å in thickness and, under cryogenic conditions, yielded 1.9 Å data [29]. The structure was solved by molecular replacement and is the first example of molecular replacement adapted and applied to an electron diffraction study. The atomic resolution density map, which for the first time in cryoEM resolved ordered water molecules, showed atomicity and a complete lipid membrane bilayer surrounding and interacting with the protein [29]. Interestingly, SCXRD studies of AQP0 were resolved albeit at lower resolution [57] and did not show the physiologically relevant 11 nm thin adhesive junctions that are prevalent in lens fiber cells [53,54]. A detailed analysis also indicated that lipids were important for membrane protein stability [6,57]. These observations underscored the need to study membrane proteins in the near-native environment of the lipid membrane and led the way for the eventual development of MicroED [22].

Following the study of AQP0, a related aquaporin from the brain, AQP4, was also solved from double-layered 2D crystals by electron diffraction in the near-native environment of the membrane. Here again, AQP4 was crystallized in its adhesive junction form [58], although the physiological relevance of this structure has not been established.

Following these two initial studies using thin 3D crystals, a third example followed suit, this time from triple-layered 2D crystals of a larger protein called connexin 26 (Cx26) [50]. Connexins are a class of membrane proteins that form cell-to-cell communicating junctions. Unlike AQP0 adhesive junctions, which do not form continuous cell-to-cell water pores, connexin junctions, known as gap junctions, do form a continuous cell-to-cell pore (Figure 2) through which small peptides and solutes can freely pass between cells. Structurally, connexins are small membrane proteins that pass the membrane as four TM helices [52] and assemble into hexamers, frequently referred to as hemichannels or connexons [51]. One hemichannel can dock with another hemichannel from the opposing membrane in a head-to-head fashion, forming what is known as a gap junction (Figure 2). Gap junctions are critical for neurotransmission and are prevalent, particularly in the brain, although most cell types express one or more types of gap junctions [52].

Crystallization of Cx26 was achieved in lipid membranes, as in the case of aquaporins. Each membrane layer contained an ordered layer of hemichannels, which spontaneously assembled into gap junctions by the addition of another membrane layer. The crystallographic symmetry allowed for three layers to assemble into a thin 3D crystal from which electron diffraction data were collected [50].

A third type of membrane junction was studied by electron diffraction, particularly after the advent of MicroED and the establishment of the methodology needed for structural studies using continuous rotation [22,26]. Claudins are membrane proteins that form tight junctions between cells, which create a watertight seal between cells [47,59]. While efforts were made at determining structures of claudins, both by SCXRD and SPA [59,60], none were able to deliver the structure of the intact tight junction. This is likely due to a common approach for both methods: an antibody fragment was used to enhance crystal contacts in the case of SCXRD and to increase the mass of the particle to enable imaging by SPA. However, this antibody fragment prevented junction formation [60]. The structure of the physiologically relevant tight junction was recently determined by MicroED of a distant member of the claudin family known as MP20 (Figure 2) [27] and is the first example of a complete tight junction structure determined experimentally.

MP20 (membrane protein of 20 kDa in size [27]) is the second most abundant lens membrane protein whose structure and function remained elusive for decades despite extensive X-ray, NMR, and single-particle attempts [27]. The full-length human MP20 structure was solved to 3.5 Å by MicroED using nanocrystals grown in LCP in which the protein was crystallized in a lipid membrane [27]. Structurally, each MP20 monomer formed 4 TM helices with its N- and C-termini located in the cytoplasm. In between TM helices 1 and 2, it contained a long extracellular loop that folded into finger-like domains exposed to the extracellular space. Likewise, an extracellular loop also connected TMs 3 and 4 on the extracellular side. MP20 assembled into tetramers, and each tetramer engaged another tetramer from the apposing membrane bilayer in a head-to-head fashion, forming a tight junction. The interactions are mainly mediated by the extracellular loops, and the entire junction spans 11 nm in thickness, reminiscent of the lens thin junctions found *in vivo* and to which MP20 localizes [27].

These examples underscore the importance of studying membrane proteins within the native environment of the lipid bilayer; however, such an approach is technically difficult and requires specialized approaches like focused ion beam milling. We recently published a step-by-step guide to FIB milling membrane protein crystals for MicroED [31]. Briefly, crystals grown in LCP tend to be inherently viscous and difficult samples for grid preparation because the solution surrounding the nanocrystals is too viscous to be blotted away easily [30,31]. We established a pipeline using fluorescence microscopy and FIB milling to target nanocrystals in LCP and expose them using FIB for MicroED [27,30]. In the case of MP20, the protein was fluorescently labeled prior to crystallization. Nanocrystals were identified by fluorescence using an integrated fluorescent microscope on the plasma FIB system. Correlative methods that we established were used to direct the FIB to mill away at the surrounding LCP and expose only a 200 nm thin crystalline lamella for MicroED. From these crystalline samples, the structure of MP20 could be determined using continuous-rotation MicroED [27]. For full details, see our guide [31].

## G protein-coupled receptors

GPCRs (also known as 7TM proteins) are a group of close to 1000 membrane proteins that form receptors to integrate signals from hormones, neurotransmitters, and other ligands into cellular responses [61,62]. GPCRs are well-known for being significant therapeutic targets [62], but determining their structure is difficult for the reasons discussed above. One of the very first structures of a GPCR was that of bacteriorhodopsin, the bacterial homologue, which was determined by electron crystallography already in the late 1970s [63].

Structurally, GPCRs contain 7TM helices and generally form a complex with a G protein in the cytoplasm [62]. Most signaling occurs via the activation of the G protein, although alternative signaling pathways have also been recently described [64,65]. Therefore, to be able to study the native receptor before it becomes committed to signaling via the G protein, it is important to study the receptor alone [62]. These receptors tend to be small, typically ∼40 kDa, meaning they are too small to study by SPA [12,13] without the attached G protein. Indeed, several studies were reported using SPA but always in the context of the G protein complex [66,67]. Studying the receptor alone is squarely the domain of crystallographic approaches, and most SCXRD studies were completed using nanocrystals grown in LCP [30] to stabilize the protein in the native environment of the lipids [6].

To date, two human GPCR proteins have been determined by MicroED; one is a standard protein used for method development [33], while the other is the vasopressin 1B receptor, a novel GPCR structure that had remained inaccessible to other structural biology methods despite extensive efforts by several laboratories [34] (Figure 3).

The human adenosine A2a receptor (A2a) was the first GPCR structure to be solved using MicroED [33]. A2a plays a role in neurological and cardiovascular functions, and it is also a target of caffeine in the brain [68]. Microcrystals of A2a bound to an antagonist (ZM241385) were grown in LCP and studied by MicroED to atomic resolution following treatment by FIB milling as outlined above [33]. Structurally, A2a adopted the canonical 7TM architecture of GPCRs with a clear density for the antagonist in the binding pocket and several unique cholesterol molecules on the surface of the protein [33]. In fact, since the protein was crystallized in LCP, a near-complete lipid bilayer was observed surrounding and interacting with the receptor [33]. A subsequent study established a more robust pipeline using FIB milling of crystals directly within the LCP, allowing MicroED data collection without sponge-phase conversion and yielding the A2a structure at 2.0 Å resolution from a single nanocrystal [30]. The resolution obtained by MicroED was the same as that obtained by others using SCXRD and SFX [67,68]. The main difference was in the sample requirements: while both SCXRD and SFX used rather large crystals up to 30 µm^3^, SFX required milliliters worth of crystals for the structure solution, while only a single 1 µm^3^ crystal was needed for MicroED [33]. This example illustrated the power of MicroED in elucidating membrane protein structure using vanishingly small crystals and minute amounts of material, indicating that samples that were unattainable by SCXRD or SFX might be amenable for structure determination by MicroED. This was indeed demonstrated with another long-sought-after GPCR, the human vasopressin 1B receptor (V1BR) (Figure 3) [34].

The vasopressin 1B receptor (V1BR) is an important pharmacological target, as it is mainly expressed in the anterior pituitary and involved in stress and mood modulation and aggression (Figure 3) [70]. Despite serious efforts from several laboratories spanning decades of research, the structure of V1BR remained elusive. This is mainly owing to the fact that V1BR crystals were too small for SCXRD and too sparse for SFX. However, they were ideal samples for MicroED [34]. V1BR was crystallized in LCP, and its structure was solved at ∼3.2 Å using MicroED [34]. A full dataset was created by merging MicroED data from 14 FIB-milled crystalline lamellae [34]. The resulting structure revealed the seven-helix bundle of V1BR and provided insights into its ligand-binding pocket and differences from related receptors. The present study represented a milestone, demonstrating MicroED's ability to determine a previously unknown GPCR structure, one that was beyond the reach of other structural biology methods [34].

## Ion channel dynamics

Ion channels are membrane proteins that regulate ion levels and signaling. These include channel proteins that permit ions like sodium, potassium, calcium, and magnesium to pass through membranes, establish proton gradients, propagate action potentials, and regulate muscle cell functions [71,72]. Generally, ion channels allow ions to pass down their concentration gradient, although in some classes energy may be used, like in the case of pumps like the P-type ATPases.

The first membrane protein structure solved by MicroED was a P-type ATPase: the Ca^2+^-ATPase pump from skeletal muscle sarcoplasmic reticulum (also known as SERCA1) (Figure 4) [20]. This protein forms thin 2D crystals that then assemble into 3D crystals and have been under investigation by electron diffraction for decades [20,73]. Early studies demonstrated that high-quality electron diffraction could be obtained from cryogenically preserved samples of the Ca^2+^-ATPase; however, a structure was never determined from such data because the intensities were inaccurate [74,75]. It was not until the advent of continuous rotation MicroED that the Ca-ATPase was determined by electron diffraction, at a modest 3.4 Å resolution [20]. This was a significant accomplishment since it showed that electron diffraction could be used to structurally solve even larger (∼110 kDa) membrane proteins. The density map of the ATPase was of sufficient quality to build an atomic model and even to discern two bound Ca^2+^ ions in the transmembrane Ca-binding sites, along with their charge-density distributions [20]. This ability to directly observe the electrostatic potential of ions was a unique benefit of MicroED Coulomb potential maps [20] and provided new insights into the pump’s E1 conformational state and ion coordination [20]. Despite this achievement, Ca-ATPase was studied previously by SCXRD at better resolution [75]; however, it demonstrated MicroED’s ability to overcome technical difficulties that have plagued the field of electron diffraction for decades.

The first novel ion channel structure determined by MicroED was that of the voltage-dependent anion channel (VDAC), a β-barrel ion channel in the mitochondrial outer membrane that regulates metabolite flux and ion exchange between the mitochondria and the cytosol [41] (Figure 4). While VDAC has been studied structurally by SCXRD previously [76–79], the functional mutant remained unattainable because the crystals were vanishingly small and could not grow larger despite serious effort. However, the sample was ideally suitable for MicroED.

MicroED was used to determine the structure of the murine mVDAC1 functional mutant of VDAC using crystals grown in bicelles [41]. The crystals were almost invisible on a cryo-EM grid and unsuitable for traditional X-ray diffraction because they were submerged in viscous bicelles and only grew as extremely thin plates [41]. After milling these plate-like crystals into lamellae suitable for MicroED using cryo-FIB milling, data were recorded and the structure solved [41]. VDAC folded into the canonical 19-stranded β-barrel arrangement, forming a large ion pore pathway at its core with the protein N-terminal α-helix folded into the pore, acting as a gate. While the structure was reminiscent of structures of VDAC solved by SCXRD [77], density for a nearly complete lipid bilayer was observed surrounding VDAC and mediating crystal contact [41].

The ion channel studies illustrated above demonstrated the ability of MicroED to discern ions, but the real power of the technique is rooted in its ability to determine structures rapidly in high throughput using vanishingly small crystals to atomic resolution. A recent study, using the non-selective ion channel NaK [42], illustrated the use of automated high-throughput MicroED approaches to not only determine the atomic resolution structure of an ion channel [46] but also to experimentally visualize channel dynamics [42] (Figures 1 and 4).

NaK is a small ion channel that contains the minimal components needed to assemble a selectivity filter within the membrane (Figure 4) [80]. Like larger ion channels, it assembles into a tetramer with the selectivity filter lining the four-fold axis of the assembly. While the structure of NaK was already determined by SCXRD [80], it was used in MicroED to probe channel dynamics. Indeed, even in the first MicroED report of NaK, a previously unseen functional state was captured: a partially hydrated sodium ion was observed at the entrance of the selectivity filter of NaK [46]. This transient state was predicted for sodium channels decades prior, but it was not captured structurally until the MicroED study was completed.

Following this initial report on NaK, high-throughput methods were established [42] by which hundreds of nanocrystals can be targeted, diffracted, and structures solved automatically [42]. This approach, which was initially established using small molecules [81], was applied to NaK, and channel dynamics were experimentally observed [42]. Several structures of NaK and its mutant NaK2CNG were determined, and the positions of the Na^+^ ions in the pore were cataloged [42]. These structures allowed an experimental determination of several distinct ion binding sites in the selectivity filter and identified channel gating and dilation mechanisms (Figure 4).

## Perspectives

- MicroED is a relatively new field of research, as the method was only established over a decade ago [22,26]. During this period, several hurdles were crossed, and the method gained momentum, especially in small molecule research and drug discovery [14,18,82,83]. MicroED already proved itself as a powerful method for delivering structures for samples that remained beyond the reach of SCXRD, SFX, and even SPA using vanishingly small crystals and with little material to atomic resolution. While the approaches described here focused on membrane proteins, the same can be done for soluble proteins, for example, mapping dynamics in enzymes as illustrated recently [42,45].
- When it comes to membrane proteins, the importance of the lipid bilayer cannot be understated or underestimated—with several examples, the data appears consistent—membrane proteins should be studied in the near native environment of the lipid bilayer so that physiologically relevant functional states can be probed. And with high-throughput methods, MicroED can even probe dynamics [42].
- As additional facilities are established, access to MicroED technology will increase, allowing investigators to find answers to questions that up until recently remained beyond the reach of other structural biology methods.

## Open Access

Open access for the present article was enabled through an open access agreement between the Biochemical Society/Portland Press and the University of California.

## Abbreviations

A2a: A2a receptor
AQP0: aquaporin-0
cryoEM: electron cryomicroscopy
cryo-FIB: cryo-focused ion beam milling
Cx26: connexin 26
GPCRs: G protein-coupled receptors
LCP: lipidic cubic phase
MicroED: Microcrystal Electron Diffraction
SCXRD: single-crystal X-ray diffraction
SFX: serial femtosecond crystallography
SPA: single-particle analysis
TEM: transmission electron microscope
V1BR: vasopressin 1B receptor
VDAC: voltage-dependent anion channel
